# The PsyCoLaus study: methodology and characteristics of the sample of a population-based survey on psychiatric disorders and their association with genetic and cardiovascular risk factors

**DOI:** 10.1186/1471-244X-9-9

**Published:** 2009-03-17

**Authors:** Martin Preisig, Gérard Waeber, Peter Vollenweider, Pascal Bovet, Stéphane Rothen, Caroline Vandeleur, Patrice Guex, Lefkos Middleton, Dawn Waterworth, Vincent Mooser, Federica Tozzi, Pierandrea Muglia

**Affiliations:** 1 Department of Psychiatry, CHUV, Lausanne, Switzerland; 2 Department of Medicine, Internal Medicine, CHUV, Lausanne, Switzerland; 3 Department of Psychiatry, University Hospital of Geneva, Geneva, Switzerland; 4 Division of Neurosciences and Mental Health, Imperial College, London, UK; 5 Medical Genetics, GlaxoSmithKline, Philadelphia, Pennsylvania, USA; 6 Genetics Division, Drug Discovery, GlaxoSmithKline R&D, Verona, Italy

## Abstract

**Background:**

The Psychiatric arm of the population-based CoLaus study (PsyCoLaus) is designed to: 1) establish the prevalence of threshold and subthreshold psychiatric syndromes in the 35 to 66 year-old population of the city of Lausanne (Switzerland); 2) test the validity of postulated definitions for subthreshold mood and anxiety syndromes; 3) determine the associations between psychiatric disorders, personality traits and cardiovascular diseases (CVD), 4) identify genetic variants that can modify the risk for psychiatric disorders and determine whether genetic risk factors are shared between psychiatric disorders and CVD. This paper presents the method as well as sociodemographic and somatic characteristics of the sample.

**Methods:**

All 35 to 66 year-old persons previously selected for the population-based CoLaus survey on risk factors for CVD were asked to participate in a substudy assessing psychiatric conditions. This investigation included the Diagnostic Interview for Genetic Studies to elicit diagnostic criteria for threshold disorders according to DSM-IV and algorithmically defined subthreshold syndromes. Complementary information was collected on potential risk and protective factors for psychiatric disorders, migraine and on the morbidity of first-degree relatives, whereas the collection of DNA and plasma samples was already part of the original CoLaus survey.

**Results:**

A total of 3,691 individuals completed the psychiatric evaluation (67% participation). The gender distribution of the sample did not differ significantly from that of the general population in the same age range. Although the youngest 5-year band of the cohort was underrepresented and the oldest 5-year band overrepresented, participants of PsyCoLaus and individuals who refused to participate revealed comparable scores on the General Health Questionnaire, a self-rating instrument completed at the somatic exam.

**Conclusion:**

Despite limitations resulting from the relatively low participation in the context of a comprehensive and time-consuming investigation, the PsyCoLaus study should significantly contribute to the current understanding of psychiatric disorders and comorbid somatic conditions by: 1) establishing the clinical relevance of specific psychiatric syndromes below the DSM-IV threshold; 2) determining comorbidity between risk factors for CVD and psychiatric disorders; 3) assessing genetic variants associated with common psychiatric disorders and 4) identifying DNA markers shared between CVD and psychiatric disorders.

## Background

### 1. Clinical and epidemiological findings on the association between psychiatric disorders and cardiovascular diseases

Both cardiovascular disease (CVD) and psychiatric disorders are major public health issues which lead to increased mortality and disability. Epidemiological studies based on structured diagnostic interviews have consistently documented high lifetime prevalence of psychiatric disorders [1-7] with even higher rates in more recent surveys. Several of these studies [8-10] as well as research in primary care settings [11] have also revealed a substantial proportion of individuals that have mood or anxiety symptoms not meeting diagnostic criteria for corresponding disorders. Although, clinical and a small number of epidemiological studies have supported the clinical significance of these syndromes [10,12-15] there is still an ongoing debate on whether or not these syndromes require treatment [16].

The bulk of research focusing on associations between depressive symptoms or disorders and CVD has documented increased prevalence of depression (ranging from 16% to 23%) among patients with various manifestations of coronary heart diseases (CHD), including myocardial infarction (MI), unstable angina, stable coronary artery disease, congestive heart failure and coronary catheterization or angioplasty. The presence of depression in patients with established CHD was found to be a predictor of poor course with increased mortality (reviews: [17-19]). Conversely, population-based prospective studies on individuals with depression or depressive symptoms have documented increased cardiovascular morbidity and mortality in these individuals, thereby implicating depression as an independent risk factor in the pathophysiologic progression of CVD, rather than merely a secondary emotional response to the illness [18,20-23]. Other symptoms and disorders investigated for their association with CVD were anxiety (review: [24]), heavy drinking (review: [25]) and personality traits [26-33].

However, the existing studies on potential associations between psychiatric disorders and CVD have suffered from serious methodological limitations, which are also likely to account for the large body of conflicting findings. These methodological limitations include: 1) the use of clinical rather than epidemiological samples (risk of treatment-seeking bias); 2) the lack of a comparison group; 3) the application of psychiatric scales for a single psychiatric syndrome rather than structured diagnostic interviews; 4) the assessment of the incidence of CVD (or risk factors for CVD) by interview techniques rather than by physical examinations, biological measures and the use of medical records; 5) the lack of assessing both CVD and risk factors for CVD, which did not allow studies to examine whether a specific psychiatric disorder was directly associated with CVD or through associations with already well-established risk factors for CVD.

### 2. Use of population sample for identifying genetic variants and biomarkers that can modify the risk for psychiatric disorders and could be shared between psychiatric disorders and CVD

Association studies represent a very powerful approach for investigating the biological basis of human diseases, comparing genotype frequencies in well-defined clinical groups to appropriate controls. However, this approach presents limits: population stratification, genetic heterogeneity and phenotype complexity affect the case-control design of genetic association studies [34]. Moreover, the real effect of a susceptibility gene and the impact of its discovery in the clinics can only be established using unselected and representative population samples, which allow for estimating prevalence of gene variants and relative genotypic disease risks. In recent years, genetics has greatly advanced and large scale genome-wide association studies (GWAS) have already delivered numerous new susceptibility genes for a variety of common conditions including type 1 and type 2 diabetes, prostate and breast cancer [35-39]. Other areas are in rapid expansion with novel loci implicated in the predisposition to complex traits, such as coronary heart disease, asthma and obesity [40-42]. The CoLaus study has already contributed to GWAS successes for several somatic traits including height, LDL, obesity [42-44] and heavy smoking [45].

The clinical practice in psychiatry suffers from the lack of objective measures. The search of peripheral markers reflecting psychiatric disease state and trait, and objective read-outs of response to treatment has been under constant scrutiny for several decades and numerous candidates have been tested based on several disease pathogenetic hypotheses. Whilst biomarkers are recognized as a great need across all disease areas, the need is possibly even more important for psychiatric disorders where disease aetiology is unknown and there is a lack of objective diagnostic criteria. However, the relevance of periphery biomarkers for common psychiatric disorders remains to be demonstrated. Recent developments in proteomic and genomic approaches are expanding the number of testable hypothesis by some orders of magnitude and allowing the exploration of patterns or signatures rather than single markers (see [46] for a review). Expectations are that, with the decreasing costs of genomic and proteomic applications, the investigation of large population-based data sets will provide the opportunity to identify more homogeneous disease subtypes and investigate biomarkers, so that biomarker studies may substantiate disease sub-groups. PsyCoLaus represents therefore a great opportunity to also investigate the presence of periphery biomarkers related to behavioral traits [46].

### 3. Specific aims of the CoLaus/PsyCoLaus study

The PsyCoLaus study is based on the large epidemiological sample of the CoLaus survey, which assessed CVD risk factors and the genetic variants associated with these conditions in the general population of the City of Lausanne [47]. The specific aims of the PsyCoLaus investigation were to 1) establish the lifetime and 12-month prevalence of threshold (DSM-IV) and subthreshold psychiatric syndromes and migraine in 35 to 66 year-old residents of the city of Lausanne; 2) test the validity of postulated definitions for subthreshold psychiatric disorders, and especially mood and anxiety syndromes as well as the concept of atypical depression using comorbidity patterns, risk of suicidal attempts, health service use, social functioning (Global Assessment of Function scores, GAF) and family history as validator variables; 3) determine the association between the lifetime history of major depressive disorder (MDD), and other psychiatric disorders and risk factors for CVD; and 4) identify genetic variants and biomarkers that can modify the risk for various psychiatric disorders and for comorbid CVD and psychiatric disorders.

## Methods

### 1. CoLaus and PsyCoLaus

PsyCoLaus is a psychiatric study conducted in a population-based cohort assessed for cardiovascular risk factors (CoLaus) (see [47] for detailed description). In brief, the CoLaus study, which was based on a sample of 6,738 individuals randomly selected from the list of residents of the city of Lausanne (Switzerland), assessed CVD risk factors and collected DNA and plasma samples for the study of genetic variants and biomarkers. Lausanne is the 5th largest city of Switzerland, localized in the French speaking part of the country. Foreigners mostly from other central European countries represent about a third of the population of Lausanne. This proportion is comparable to that of other Swiss cities, but higher than the average of approximately 20% in the whole country. Compared to other European countries, the Swiss population is relatively stable favoring the completion of prospective follow-up studies, such as the Zurich cohort study, over decades [48].

The present study (PsyCoLaus), based on the CoLaus sample, included a semi-structured diagnostic interview and a number of self-rating instruments that evaluated personality traits, attitudes, functioning and sleep patterns.

### 2. Participants

The Institutional Ethic's Committee of the University of Lausanne approved the CoLaus and subsequently the PsyCoLaus study. All participants signed a written informed consent after having received a detailed description of the goal and funding of the study.

The recruitment and medical assessment of the CoLaus sample, which was completed between 2003 and 2006, has been described in detail [47]. The random sampling procedure was based on a complete list of the Lausanne inhabitants aged 35–75 years (n = 56,694 in 2003), provided by the population register of the city. Of the initial 19,830 subjects sampled, 54 subjects were considered as non-eligible before contact and 15,109 (76%) responses were obtained. Among responders, 6,189 (41%) subjects refused to participate and 799 (5%) were considered as non-eligible (moved away, out of the age range or deceased). The sample of 8,121 subjects who agreed to participate represented 41% of the initially sampled population and 57% of all eligible responders. Among these subjects, 6,738 completed the examination (6,188 Caucasians and 549 Non-Caucasians), whereas 1,383 could not be included into the study despite their will to participate because the number of subjects who agreed to participate was higher than the number of subjects initially planned for the CoLaus study (one additional subject withdrew after consent).

All 35 to 66-year old subjects of the CoLaus sample (n = 5,535), were invited by letters to also participate in the psychiatric evaluation. Those who did not respond to the letter were contacted by phone. All subjects who were sufficiently fluent in French or English and agreed to participate were included into the PsyCoLaus sub-study and underwent the psychiatric assessment between 2004 and 2008.

### 3. Clinical assessments

Assessment within the CoLaus study [47] included the collection of socio-demographic, personal and treatment history data as well as family history information of CVDs (myocardial infarction, stroke and coronary artery disease) and their risk factors. In women, further data regarding reproductive and obstetrical history, oral contraception and hormonal replacement therapy was collected. The somatic exam encompassed measurements of body weight, height, blood pressure (triplicate measure three times on the left arm after at least a 10-minute rest in the seated position), heart rate (triplicate measure), waist and hip circumferences, fat and fat-free mass assessed by electrical bioimpedance [47]. Moreover, venous blood samples were drawn from each participant after an overnight fast, in order to measure the levels of glucose, LDL-cholesterol, HDL cholesterol, and triglycerides. A random subgroup also performed an oral glucose tolerance test. A urine sample was collected for the assessment of creatinine and albumin. Finally, participants completed the 12-item General Health Questionnaire (GHQ-12; [49]; French translation: [50]). This self-rating instrument was specifically developed to detect the presence of minor psychiatric symptoms. In a study including 25,916 patients in 15 countries, the GHQ was found to work as well as the longer 28-item version of the instrument [51]. According to the Likert scoring method, a threshold score of 12 revealed a sensitivity of 78.9% and a specificity of 67.4% to detect psychopathology.

Within the PsyCoLaus sub-study, diagnostic information was collected using the Diagnostic Interview for Genetic Studies (DIGS, [52]). The DIGS was developed by the NIMH Molecular Genetics Initiative in order to obtain a more precise assessment of phenotypes through 1) a semi-structured design corresponding to a wide spectrum of DSM-IV Axis I criteria and suicidal behavior, and 2) the collection of extensive information on the course and chronology of comorbid conditions. An updated version of the DIGS [53] includes DSM-IV criteria. The French translation of the DIGS [54] resulted from a collaborative effort between the Department of Psychiatry of Lausanne and the INSERM in Paris. Several modifications were incorporated into the French version: 1) a screening question was added to the mania section to lower the threshold for entering the chapter by asking whether friends or family members had observed episodes where the subject's mood was more elated than normal; 2) additional questions were added to the depression section in order to elicit criteria for atypical depression features (leaden paralysis, long-standing patterns of interpersonal rejection sensitivity, mood reactivity) and recurrent brief depression (maximal number of episodes within a 12-month period); 3) a section on generalized anxiety disorder (GAD) was added using the questions from the Schedule for Affective Disorders and Schizophrenia – Lifetime Version (SADS-L, [55]); 4) the brief phobia chapter of the DIGS was replaced by the corresponding more extensive chapters from the SADS-L; and 5) the original DIGS section on nicotine consumption was largely extended to elicit DSM-IV abuse and dependence criteria. As long as a subject was treated in a psychiatric setting in Switzerland, personal history information was completed by the collection of medical records in order to obtain supplementary data on symptoms, impairment, duration, timing of illness and treatment. The applied semi-structured interview allowed for the establishment of lifetime and 12-month prevalence of a large array of specific DSM-IV axis-I (threshold) disorders as well as algorithmically-defined subthreshold mood and anxiety syndromes according to [8,9]. The French version of the DIGS revealed excellent inter-rater reliability in terms of kappa and Yule's Y coefficients for major mood and psychotic disorders [56] and substance use disorders [57], whereas the 6-week test-retest reliability was slightly lower [56,57].

Additional data collection based on interview techniques included headache symptoms ('Diagnostic Interview for Headache Syndromes' DIHS), life-events (short interview of F. Amiel-Lebigre; [58]) and family history information. Family history information was gathered using the modified version of the Family History-Research Diagnostic Criteria (FH-RDC; [59], as initially used in the Yale Family Study [60]. This version (adaptation to DSM-III-R and DSM-IV) was translated into French by our group, who undertook extensive validation efforts of this tool by establishing the agreement and prevalence estimates between this instrument and semi-structured interviews for a series of specific psychiatric diagnoses [61,62]. Generally, the family history method revealed high specificity but low sensitivity.

Complementary information on personality and temperamental features, familial functioning, coping and sleep were obtained using a self-report battery including the following instruments: the State-Trait Anxiety Inventory (STAI; [63,64]), the Retrospective Self Report Childhood Inhibition (RSRCI; [65]), the Dimensions of Temperament Survey Revised (DOTS-R; [66]), the Eysenck Personality Questionnaire (EPQ; [67,68]), the Type A behavior [69,70], the Sensitivity to Reward (STR), the Parental Bonding Instrument (PBI; [71-73]), the Family Adaptability and Cohesion Scale III (FACES III; [74,75]), the Dyadic Adjustment Scale (DAS; [76,77]), the Family Attitude Scale (FAS-30; [78]), the Euronet: Problem Resolution Strategy [79] and the MOS-Sleep Module [80].

### 4. Genotyping and biological data

During the CoLaus evaluation, participants donated blood after a 12-hr fasting period for clinical chemistry and genetic analyses. Most of the assays were performed by the Clinical Chemistry Laboratory within the Lausanne University Hospital. Plasma, serum and RNA are available for biomarkers studies. Nuclear DNA was extracted from whole blood for whole genome scan analysis and genome-wide genotyping was performed on all the 6,188 participants to the CoLaus, using the Affymetrix 500 K SNP chip. Participants were removed from the analysis on the basis of the following sample quality control criteria: any participant whose sex was inconsistent with genetic data from X-linked SNPs; the proportion of genotypes called was less than 90%; having inconsistent genotypes when compared with duplicate samples. In total, 5636 participants remained after sample quality control exclusions. We then applied SNP exclusions with the following criteria: SNPs that were monomorphic among all samples; SNPs with genotypes on less than 95% participants; SNPs that were out of Hardy-Weinberg equilibrium (p < 1·0 × 10^-7^). After these quality control procedures, 370 697 SNPs remained for analysis [43].

The inflation factor (λ), which was estimated from the mean of the χ^2 ^tests generated on all SNPs that were tested, was calculated to be 1,010. This lambda value, which is very close to 1, indicates the absence of major population structure, i.e. that the sample is quite homogeneous genetically [81].

### 5. Data management and quality control

Interviewers were required to be psychologists or psychiatrists, who were trained over a two-months period. Their training included rating tapes and supervised co-ratings. In order to provide ongoing supervision throughout the study, each interview and diagnostic assignment was reviewed by an experienced senior psychologist.

Phenotypic data were entered into a secured, internet-based database. The database was designed to confirm the validity of the identification codes, establish the completeness of the information keyed in and to perform basic data checks. All discrepancies were recorded in a case report form kept in a locked room. All modifications of the data were automatically recorded, including the identity of the investigator who made each modification, the date, the old and the new values.

### 6. Precision of prevalence assessment and power estimates

Within the whole sample, disorders such as schizophrenia or bipolar-I disorder with an expected lifetime prevalence of 1% can be estimated with 95% confidence within a range of +/- 0.31% (i.e. the lower and upper bounds of a 95% confidence interval for a disorder with a 1% prevalence would be 0.69% and 1.31%). For more common disorders, such as MDD, with an expected lifetime prevalence of 15%, the prevalence can be estimated within a range of about +/- 1.1% in the whole sample and within a range of about +/- 4% in a 5-year age-sex stratum.

The power for the analysis of associations between disorders and dichotomous variables is provided on Table 1 according to the formula for dichotomous variables [82] and assuming a two-tailed p-value of 0.05. Even for relatively rare disorders or syndromes with a prevalence of 1% (bipolar disorder or schizophrenia) an association with correlates present in 25% of the sample (e.g. 25 highest percentile regarding triglycerides or cholesterol levels) could be detected with a probability of 63% if the relative risk is 2 and already 88% if the relative risk is 2.5. However, typical psychiatric disorders such as MDD documented to be associated with risk factors for CVD have prevalence rates of 10% or more. For such conditions, a 2 times increased risk with respect to a correlate present in 5% of the sample (e.g. diabetes) could be detected with a probability of 97%, whereas a 1.5 times increased risk could be detected with a probability of 81% for correlates present in 10% of the sample (e.g. high blood pressure).

**Table 1:** Power for the analysis of the associations between two dichotomous variables (%)

**Prevalence of the index disorder**	**Relative risk for the presence of the dichotomous correlate in individuals with the index disorder**	**Frequency of dichotomous correlates**									
		
		**1%**	**3%**	**5%**	**10%**	**15%**	**25%**	**50%**	**75%**	**90%**	**95%**
**1%**	**1.5**	9	12	14	18	21	26	28	18	8	5
	
	**2**	17	25	31	43	52	63	70	52	21	9
	
	**2.5**	25	39	49	66	77	88	93	83	41	16

**3%**	**1.5**	12	19	25	37	46	58	67	51	23	12
	
	**2**	26	45	59	80	90	97	99	97	70	36
	
	**2.5**	40	68	83	96	99	100	100	100	96	69

**5%**	**1.5**	15	26	35	53	65	79	88	76	41	20
	
	**2**	34	61	78	94	98	100	100	100	93	64
	
	**2.5**	53	85	95	100	100	100	100	100	100	94

**10%**	**1.5**	21	42	57	81	91	98	100	98	76	46
	
	**2**	51	87	97	100	100	100	100	100	100	96
	
	**2.5**	77	99	100	100	100	100	100	100	100	100

**15%**	**1.5**	28	57	75	94	99	100	100	100	94	69
	
	**2**	67	97	100	100	100	100	100	100	100	100
	
	**2.5**	91	100	100	100	100	100	100	100	100	100

**20%**	**1.5**	35	71	88	99	100	100	100	100	99	86
	
	**2**	80	100	100	100	100	100	100	100	100	100
	
	**2.5**	98	100	100	100	100	100	100	100	100	100

**25%**	**1.5**	42	82	95	100	100	100	100	100	100	95
	
	**2**	91	100	100	100	100	100	100	100	100	100
	
	**2.5**	100	100	100	100	100	100	100	100	100	100

For genetic analyses, power calculations were done using the program Genetic Power Calculator ([83]; ).

We have estimated that for a dichotomous trait such as recurrent MDD – that has a prevalence of around 15% in the PsyCoLaus cohort – the study has approximately 85% power to detect an allele with 50% allele frequency that has a genotypic relative risk = 3 under a dominant model (type 1 error rate of 10^-7 ^taking into account 500'000 genetics markers). For a continuous behavioral trait such as Neuroticism that has been measured in our cohort we would have power of approximately 99% to detect additive QTL effect that explains 2% of the variance (sample size = 3000; type 1 error rate of 10^-7 ^taking into account 500'000 genetics markers); power drops rapidly for smaller effect (i.e. < 2%). However, this large genotypic relative risk of 3 is unlikely to exist for common traits, including psychiatric disorders. Therefore, the sample size in our study does not provide enough statistical power to detect SNPs with small effects. In order to increase the power to detect such SNPs, the sample needs to be combined with those of similar studies [84].

## Results

### 1. Recruitment and sociodemographic characteristics of the sample

Sixty-seven percent of the participants of the CoLaus study in the age range between 35 and 66 years accepted the psychiatric evaluation, which resulted in a sample of 3,691 individuals who underwent both the medical and psychiatric exam. Ninety-two percent of them were Caucasians. The gender distribution of the PsyCoLaus sample (46.9% males) did not differ significantly from that of the general population in the same age range, but the youngest 5-year band of the cohort was underrepresented and the oldest 5-year band overrepresented (Table 2). Table 3 provides socio-demographic characteristics of the sample. The mean age of the participants was 49.6 years (s.d. 8.8 years) at the CoLaus and 50.9 (s.d. 8.8 years) years at the psychiatric exam. With a representation of 70.7%, Swiss citizens were over-sampled as their proportion is only 67.8% in the whole population of the city of Lausanne within the same age range. Two thirds of the males but only about half of the females were living with a partner, whereas more than a quarter of females and a sixth of males were separated or divorced. The educational level was higher in males than in females. Similarly, the proportion of professionally active people was larger in males than females. The main difference resulted from the fact that 33.2% of females were housewives, whereas only 2.4% of males undertook the role of housekeepers. Among the professionally active persons, the mean degree of professional activity was 95.4% in males and 74.4% in females.

**Table 2:** Age and sex distributions of the sample

Age	Recruited sample (n = 3691)			Difference recruited vs. intended sample according to the distribution in the general population (%)		
	Males	Females	All	Males	Females	All

35–39	322	327	649	-19.1	-14.6	-16.9
40–44	337	343	680	-2.8	-3.0	-2.9
45–49	321	333	654	6.2	7.1	6.7
50–54	250	319	569	0.5	11.7	6.5
55–59	217	287	504	-11.8	0.1	-5.4
60–66	283	352	635	22.3	18.2	20.0

35–66	1730	1961	3691	-2.4	2.3	0.0

Sex: χ^2 ^= 1.0; df = 1; p = n.s.

Age: χ^2 ^= 25.4; df = 5; p = < 0.0001

**Table 3:** Socio-demographic characteristics of the PsyCoLaus sample

	Overall (n = 3691)	Males (n = 1730)	Females (n = 1961)
Race (%)			
Caucasians	92.1	92.7	91.6
Age (mean, s.d.)			
Somatic exam	49.6 (8.8)	49.2 (8.8)	50.0 (8.8)
Psychiatric exam	50.9 (8.8)	50.5 (8.8)	51.3 (8.8)
Citizenship			
Swiss	70.6	66.8	74.0
Marital status (%)			
Single	15.6	14.6	16.5
Married/cohabitation	58.6	67.0	51.1
Divorced/separated	22.7	17.3	27.5
Widowed	3.1	1.2	4.8
Education (%)			
Basic	16.1	14.0	17.9
Apprenticeship	37.4	37.5	37.4
High school/college	18.6	15.0	21.7
University	28.0	33.6	23.1
Work status (%)			
Professional	57.3	75.1	41.6
Unemployed	3.1	3.8	2.4
Retired	9.2	7.7	10.6
Disabled/sick	8.8	9.1	8.4
Other	21.7	4.2	37.0

### 2. Prevalence of selected cardiovascular risk factors

The prevalence of somatic cardiovascular risk factors such as obesity (BMI ≥ 30 kg/m^2^), hypertension (systolic BP ≥ 140 or diastolic BP ≥ 90 mmHg or current treatment for hypertension), diabetes (fasting blood glucose ≥ 7 mmol/l or current treatment with oral hypoglycemic agents or insulin) and dyslipidemia (HDL-cholesterol <1.0 mmol/l or triglycerides ≥ 2,2, mmol/l or LDL cholesterol ≥ 4.1 mmol) was 13.5%, 28.7%, 5.5% and 32.0%, respectively. Except for diabetes, these rates were slightly lower than those in the CoLaus sample (14.8%, 31.3%, 5.5%, 33.8%, respectively), indicating that individuals exhibiting obesity, hypertension and dyslipidemia were slightly less prone to participate at the psychiatric evaluation than those without these cardiovascular risk factors.

### 3. General Health Questionnaire (GHQ) data

Among the 5,230 participants of CoLaus aged between 35 and 66 years 5,020 responded to all questions of the GHQ-12 (96.0%), which allowed for an estimation of potential selection bias due to non-participation in PsyCoLaus. However, GHQ-12 scores between participants and non-participants of the psychiatric exam did not differ significantly (z = 1.92; p = n.s.). The mean scores according to the Likert method were 11.11 (s.d = 4.63) and 10.87 (s.d. = 4.60), respectively. Moreover, after adjustment for multiple testing according to Bonferroni, GHQ-12 scores did not differ according to the presence or absence of the somatic cardiovascular risk factors obesity, hypertension, diabetes or dyslipidemia. This lack of association was observed in both the participants and non-participants of PsyCoLaus.

### 4. Family history information and additional phenotypic data collection

A total of 23,238 family history reports could be collected from 3,310 subjects (mean: 7 records per subject). These reports included 6,558 reports on parents, 7,501 on siblings, 517 on half-siblings, 4,984 on children and 3,462 on spouses. Moreover, according to the DIGS interview 892 respondents (24.2%) reported that they suffered from headache. Consequently, these symptoms were investigated in detail using the DIHS. Finally, 71.6% of the participants completed the self report battery.

## Discussion

### 1. Suitability of the study design

The herein presented PsyCoLaus study combines an investigation of cardiovascular risk factors with a comprehensive psychiatric assessment and the collection of DNA in individuals recruited from the general population. The large majority of previous research on associations between psychiatric disorders and CVD included depression rating scales rather than diagnostic interviews. Only four studies (two of them based on the same sample) elicited criteria for depression using structured diagnostic interviews. However, in these studies most data on somatic risk factors for CVD were collected using reports from study subjects (see additional file 1: Table 4), which entailed the risk of inaccurate information and affected the ability to accurately adjust for them in the analyses. Moreover, all these studies focused on depression only and did not determine the effects of potential comorbid conditions such as anxiety disorders. In addition, none of these studies included genotype assessment.

**Table 4:** Studies of associations between psychiatric disorders and cardiovascular diseases including diagnostic interviews for psychiatric disorders

	Sample					Assessed cardiovascular risk factors			
					
Study authors	Target population and Age at baseline (years)	N *(males/* *females)*	Diagnostic interview for psychiatric disorders	Psychiatric disorders analyzed	Outcome measure	Socio- demographic variables	Measured medical variables	Self-reported variables	Genetic testing
Aromaa *et al*. (1994)	Finnish adults > 40	3811 (1825/1986)	Present State Examination (PSE)	Depression	Fatal cardio-vascular disease	Age			No
Pratt *et al*. (1996)	US adults (ECA study, Baltimore) > 18	1551* (583/968)	Diagnostic Interview Schedule (DIS)	Depression Dysphoria	Non-fatal myocardial infarction	Age Sex Marital status		Hyper-tension	No
Larson *et al*. (2001)	US adults (ECA study, Baltimore) > 18	1703* (632/1071)	Diagnostic Interview Schedule (DIS)	Depression Dysthymia	Stroke (fatal and non fatal measures combined)	Age Sex Education		Diabetes Blood-pressure Heart-problems Smoking	No
Penninx *et al*. (2001)	Dutch older adults (LASA study) 55–85	2847 (1367/1480)	Diagnostic Interview Schedule (DIS)	Major depression Minor depression	Fatal cardio-vascular disease	Age Sex Education	Hyper-tension BMI	Diabetes Stroke Lung-disease Cancer Smoking Alcohol	No
Current study	Swiss urban adults (CoLaus/PsyCoLaus) 35–66	3691 (1730/1961)	Diagnostic Interview for Genetic Studies (DIGS)	Depression Anxiety disorders Substance use disorders	Coronary heart- disease, stroke	Age Sex Education Marital status	Hyper-tension Diabetes Dyslipi-demia BMI	Smoking Alcohol Physical- activity	Yes

* = CVD free population at entry

The CoLaus/PsyCoLaus design has attempted to overcome a series of limitations of previous research that focused on associations between psychiatric disorders and risk factors for CVD. Besides the use of a sample recruited in the general population, which should prevent the risk of Berkson's bias and minimize the problem of inappropriate comparison groups, the application of a semi-structured psychiatric interview and a thorough somatic investigation including also blood chemistry measures ensures the collection of valid data on both psychiatric disorders and risk factors for CVD. The simultaneous assessment of a large array of DSM-IV axis-I disorders also allows for the identification of specific psychiatric disorders which are most strongly associated with risk factors for CVD. In contrast to the bulk of previous population-based research in psychiatry, the applied semi-structured interview also enables the assessment of algorithmically-defined mood and anxiety syndromes below the level of DSM-IV and of their clinical impact.

There is general consensus among geneticists and genetic epidemiologists [85-89] on the value of conducting genetic studies in large population-based association studies. Susceptibility genes for common diseases are by and large identified in clinical samples and very often in narrowly defined categories of disorders in order to increase power since most severe conditions are associated with higher genetic loading (e.g. recurrent MDD). Genetic studies in the community are essential to determine the genetic risk attributable to susceptible gene variants at a population level for both narrowly and broadly defined disorders. They also provide the opportunity to estimate a population based genotype relative risk. The PsyCoLaus study offers such a unique opportunity and, in addition, it provides the chance to identify genetic variants that may be shared risk factors for psychiatric disorders and CVD.

### 2. Limitations

Limitations mainly result from constraints regarding the sample and the cross-sectional design. Indeed, the comprehensive physical exam, blood chemistry tests including fasting glucose and DNA collection is easier to organize in an urban area with a central hospital, where the clinical and laboratory research team is localized. However, urban populations are generally not representative of the whole country, as they typically include an increased proportion of diseased subjects. This could be reflected by the mean GHQ score of the sample, which was higher than in a population-based Australian study [90]. Indeed, as GHQ scores did not differ between participants of CoLaus who accepted and those who refused the psychiatric exam, either the general population of the city of Lausanne reveals a relatively high level of psychopathology or those with increased levels of psychopathology were already more likely to participate in the original CoLaus study. The over-representation of diseased or disabled subjects in an urban region would entail the establishment of increased prevalence estimates as compared to the general population of a country, whereas the assessment of associations between psychiatric disorders and risk factors for CVD as well as genetic analyses should be at least less affected or not affected by the choice of the population. Indeed, there was no evidence for differential associations between somatic risk factors for CVD and GHQ-scores according to participation status, although individuals exhibiting these risk factors were slightly less likely to participate at the psychiatric evaluation.

The comprehensive assessment including several distinct components has certainly contributed to the relatively low participation rate. Nonetheless, the response rate of 67% for the psychiatric part was very similar to that of the EPIC-Norfolk United Kingdom Prospective Cohort Study (response rate = 72%; Surtees et al. 2008), which, however, was based on a self-assessment approach and did not include a diagnostic interview. Given the time-consuming psychiatric evaluation, it is not surprising that 35 to 39-year-old individuals with typically high levels of professional activity and familial constraints revealed the lowest and the 60 to 66 year-old mostly retired individuals the highest response rate. As specific data on the work/disability status for the general population of the city of Lausanne could not be obtained, it was not possible to test the presence of selection bias with respect to work/disability status.

The requirement to be fluent in French or English to complete the psychiatric interview has only slightly reduced the participation of foreigners. Nevertheless, morbidity estimates for specific groups of migrants could be biased in the case of an association between the level of social integration and morbidity.

Another limitation is the cross-sectional study design, which does inherently not allow us to easily distinguish between cause and consequence (temporal ambiguity) given the risk of inaccurate recall of the onset of diseases. For this reason, potential associations between psychiatric disorders and risk factors for CVD will be difficult to interpret regarding the direction of causality.

Finally, the sample size is still too small to detect SNPs with small effects as expected for psychiatric disorders. Therefore, in order to increase the power to detect such SNPs, the sample needs to be combined with those of similar studies.

### 3. Perspectives

As the sample of individuals suffering from threshold and subthreshold mood and anxiety syndromes constitutes an ideal proband group for an epidemiological family study, we will also investigate all available first-degree relatives of the PsyCoLaus sample within the next two years using the same psychiatric assessments. In the present study, probands were asked whether they would allow us to contact their first-degree family members and spouses. Such a population-based family study will allow for the testing of the generalizability of findings from existing family studies, which were based on clinical probands, and will extend the scope from DSM-IV disorders to subthreshold syndromes.

Moreover, a longitudinal follow-up of all participants of the PsyCoLaus study is planned. More than 95% of the sample has consented to be contacted for follow-up.

## Conclusion

Despite limitations, the presented study should significantly contribute to the current scientific knowledge and subsequent clinical benefice by: 1) establishing the potential clinical relevance of specific psychiatric syndromes below the threshold of DSM-IV, which will be crucial to decide whether and which of the postulated subthreshold syndromes are a public health issue requiring treatment; 2) completing a genome wide association analysis to identify DNA markers for risk factors for various psychiatric disorders; and 3) assessing the associations between risk factors for CVD and a large array of psychiatric disorders and personality traits, which should allow for the identification of specific psychiatric disorders or personality traits and genetic variants that are most strongly associated with risk factors for CVD. The better understanding of the interplay between specific psychiatric disorders, personality traits and risk factors for CVD should ultimately lead to the development of more specific and effective behavioral interventions in individuals suffering from psychiatric conditions and to a more successful prevention of CVD.

## Competing interests

Pierandrea Muglia, Federica Tozzi, Dawn Waterworth, Vincent Mooser and Lefkos Middleton were or are full-time employees of GlaxoSmithkline.

## Authors' contributions

All authors participated in the study design and conception of the project. MP and SR analyzed the epidemiological data, FT, PM the genetic data. MP, FT and PM drafted the article, which was revised by PV, PB, SR, CV, PG, VM, DW, FT, and PM. All authors read and approved the final manuscript.

## Pre-publication history

The pre-publication history for this paper can be accessed here:


